# Hydrogen Sulfide Protects Against Uremic Accelerated Atherosclerosis via nPKCδ/Akt Signal Pathway

**DOI:** 10.3389/fmolb.2020.615816

**Published:** 2021-02-09

**Authors:** Xiangxue Lu, Han Li, Shixiang Wang

**Affiliations:** Department of Blood Purification, Beijing Chao-Yang Hospital, Capital Medical University, Beijing 100020, China

**Keywords:** uremia accelerated atherosclerosis, ApoE^−/−^mice, hydrogen sulfide, nPKCδ/Akt signaling pathway, vascular cell adhesion molecule-1

## Abstract

**Background:** Cardiovascular disease is the most common complication and leading cause of death in maintenance hemodialysis patients. Previous studies have found that disorders of cystathionine-gamma-lyase/hydrogen sulfide (CSE/H_2_S) system in maintenance hemodialysis patients are correlated with the risk of cardiovascular disease. Although the role of CSE/H_2_S system in UAAS has been preliminarily explored, the molecular mechanism of CSE/H_2_S is still not systematically elaborated, and the molecular mechanism of nPKCδ and its related signaling pathway in UAAS is still not thoroughly studied.

**Methods:** Forty chronic kidney disease (CHD) patients were studied and the activation of nPKCδ in peripheral blood mononuclear cells (PBMCs) were detected. ApoE^−/−^ mice aged 6 weeks were treated with 5/6 nephrectomy and high-fat diet to make UAAS model. They were divided into Sham group (Sham group), UAAS group (UAAS group), UAAS+L-cysteine group (UAAS+L-cys group), UAAS+sodium hydrosulfide group (UAAS+NaHS group) and UAAS+propargylglycine group (UAAS+PPG group). The UAAS+L-cys group, UAAS+NaHS group and UAAS+PPG group were respectively given L-cys, NaHS and PPG by intraperitoneal injection. The aorta was taken 6 weeks after surgery. Western blot was used to detect the activation of nPKCδ, the phosphorylation of Akt, and the expression of VCAM-1 in the aorta of mice.

**Results:** The membrane translocation of nPKCδ in CHD patients with plaque was higher than that in CHD patients without plaque. The membrane translocation of nPKCδ and the expression of VCAM-1 in UAAS group was higher than sham group, L-cys or NaHS injection could suppress the membrane translocation of nPKCδ and the expression of VCAM-1, but PPG treatment resulted in more membrane translocation of nPKCδ and the expression of VCAM-1 (*P*<0.05, n=6 per group). Akt phosphorylation in UAAS group was lower than sham group, and L-cys or NaHS injection could suppress the degradation of Akt phosphorylation, but PPG treatment resulted in more decrease in the Akt phosphorylation (*P*<0.05, n=6 per group).

**Conclusion:** Endogenous CSE/H_2_S system protected against the formation of UAAS via nPKCδ/Akt signal pathway. The imbalance of CSE/H_2_S system may participate in the formation of UAAS by affecting the expression of downstream molecule VCAM-1, which may be mediated by nPKCδ/Akt signaling pathway.

## Introduction

Cardiovascular disease is the most common complication and leading cause of death in patients with chronic kidney disease, especially those on maintenance dialysis (Fishbane and Berns, 2005). The incidence age of atherosclerosis in patients with end-stage renal disease is advanced, and the disease progression is faster, which is known as uremia accelerated atherosclerosis (UAAS) (Sun et al., 2019).

The pathogenesis of atherosclerosis mainly includes vascular endothelial injury and dysfunction, monocyte infiltration, foam cell formation and vascular smooth muscle cell proliferation (Gistera and Hansson, 2017). H_2_S is a newly identified third gaseous signaling molecule after nitric oxide (NO) and carbon monoxide (CO), which has a variety of physiological regulatory functions in the cardiovascular system (Wang et al., 2017). A large number of studies have shown that H_2_S plays a protective role in the formation and progression of atherosclerosis (Peh et al., 2014; Yao et al., 2016). Given the high-fat diet ApoE^−/−^ mice supplement exogenous H_2_S donor sodium hydrosulfide (NaHS) could inhibit the progress of the blood vessel damage (Ford et al., 2013), lack of cystathionine-gamma-lyase (CSE), the key enzyme in generating H_2_S can lead to a variety of pathological changes such as vascular smooth muscle cells (VSMCs) proliferation (Yang et al., 2010), macrophages release TNF alpha (Wang et al., 2013).

Protein kinase C (PKC) is an important signal transduction molecule in cells (Singh et al., 2017). The researches have shown that nPKCδ played an important role in regulating the function of vascular endothelial cells, the formation of foam cells, and the function of VSMCs (Fan et al., 2014). Although the role of CSE/H_2_S system in UAAS has been preliminarily explored, its molecular mechanism has not been systematically elaborated, and the molecular mechanism of nPKCδ and its related signaling pathway in UAAS has not been thoroughly studied. Therefore, the purpose of this study was to determine the impact of CSE/H_2_S system and nPKCδ/Akt signaling pathway on atherosclerosis development in UAAS mice, and identify the possible molecular mechanism.

## Materials and methods

### Patients

A total of 20 CHD patients with atherosclerosis (CHD+AS) and 20 CHD patients without atherosclerosis were included in this study. The patients had no residual renal function and had undergone regular dialysis treatment for at least 3 months, without clinical evidence of heart failure, a recent acute coronary event, autoimmune disease, cancer, and active infection and taking aspirin, steroid, or immunosuppressive drugs. CHD patients with AS were defined as localized thickening of intima-media thickness (IMT) ≥1.2 mm that did not uniformly involve the whole wall of carotid artery. In control group, 20 age and gender matched healthy individuals were enrolled in this study. The fasting blood samples of HD patients were taken from the arterial end of the vascular access immediately before initiation of the mid-week HD session at baseline. The levels of albumin (Alb), alanine transaminase (ALT), aspartate aminotransferase (AST), triglycerides (TG), total cholesterol (Tch), low density lipoproteincholesterol (LDL-C), high sensitivity C reactive protein (hsCRP), creatinine (Cr), blood urea nitrogen (BUN), calcium (Ca), and phosphorus (P) were measured by standard laboratory methods using an autoanalyzer. Serum intact parathyroid hormone (iPTH) was determined by immunoradiometric assay. Peripheral blood mononuclear cells (PBMCs) were separated from blood samples by lymphocyte separation medium, which were used to detect the nPKCδ activation in vitro.

### Animals and animal models

Male ApoE^–/–^ mice at age of 5 weeks were purchased from Beijing Vital River Laboratory Animal Technology Co., Ltd (License No. SCXK (Beijing) 2016–0006) and were adaptive feeded for one week. Animals were housed in clean degree laboratory, 4/cage, with the room temperature of 18–25°C, the relative humidity of 35–50%, and sufficient oxygen. High-fat diet was purchased from Research Diets (Cat. No.: D12108C).

At the age of 6 weeks, ApoE^–/–^ mice were divided into five groups randomly depending on the treatment (n=6 per group). The sham group mice were fed with a high-fat diet and sham surgery with decapsulation of both kidneys; UAAS group mice were fed with a high-fat diet (HFD) and subjected to 5/6 nephrectomy; the UAAS group mice intraperitoneal injected with L-cys (substrate of CSE, 50 mg/kg body weight/day) were considered to the UAAS+L-cys group; the UAAS group mice intraperitoneal injected with NaHS (H_2_S donor, 56 μmol/kg body weight/day) were considered as the UAAS+NaHS group; the UAAS group mice intraperitoneal injected with PPG (CSE inhibitor, 37.5 mg/kg body weight/day) were considered to the UAAS+PPG group.

The 5/6 nephrectomy was performed in two stages under isoflurane anesthesia as previously described (Leelahavanichkul et al., 2010). Briefly, a longitudinal incision was made in the lateral skin under the left costal ridge angle of the mouse, about 1cm long. The muscle was cut layer by layer, the left kidney was exposed and the renal capsule was separated, then 2/3 of the renal tissue in the upper and lower pole of the left kidney was removed. After 2 weeks, the right kidney was exposed in the same way, and the pedicle was ligated with 3–0 nonabsorbent sutures. After the ligation was confirmed, the right kidney was removed. In the sham group, the skin was incised under anesthesia to separate the kidney, but the kidney was not excised.

All procedures were performed in accordance with the guidelines set by the recommendations in the Guide for the Care and Use of Laboratory Animals of the National Institutes of Health and approved by the experimental animal ethics committee of Beijing Chao-Yang Hospital, Capital Medical University.

### Western blot analysis

Western blot analysis was used to detected activation of nPKCδ Akt phosphorylation and VCAM-1 expression. Six weeks after intraperitoneal injection of drugs, the mice were sacrificed by cervical dislocation, the aorta was separated from the aortic root to the iliac artery bifurcation, the surrounding adipose tissue was stripped, washed with normal saline, and stored in the refrigerator at −80°C. PBMCs of patients were also stored in the refrigerator at −80°C for further western blot analysis.

The Membrane and Cytosol Protein Extraction Kit was used to extract the cytosolic and membrane proteins. PMSF was added earlier to make the final PMSF concentration of 1 mM in reagents A and B. Membrane protein extraction reagent A with PMSF was added to the aortic tissue debris, homogenized, sonicated, vortex reconstituted, then centrifuged at 700 g for 10 min at 4°C, the supernatant was collected and then centrifuged at 14000 g for 30 min at 4°C, the supernatant was collected as cytoplasmic protein. The membrane protein extraction reagent B with PMSF was added to the precipitate, homogenized, sonicated, vortex reconstituted, and centrifuged at 14000 g for 5 min at 4°C, the precipitate was collected as the membrane protein and stored at −80°C for use.

To extract the total protein, RIPA lysis buffer with protease inhibitor and phosphatase inhibitor was added to the cleaved aortic tissue, homogenized, sonicated, vortex reconstituted, then centrifuged at 12000 g for 10 min at 4°C, the supernatant was collected as total protein, stored at −80°C for use.

Protein levels of nPKCδ of cytoplasmic and membrane protein, Akt, phosphorylated-Akt (p-Akt), VCAM-1 of total protein in mice aorta were analyzed by Western blot. BCA method was used to detected the concentration of protein. 20 μg of each sample were electrophoresed on a 10% SDS-polyacrylamide gel. The protein was electrophoresed at an electric current of 30 mA. After completion of electrophoresis, the protein was transferred to polyvinylidene fluoride film (PVDF) membranes. The PVDF membranes were blocked with 10% skim milk which was formulated in Tris-HCI buffered saline solution (TBST) for 60 min, then the membrane was washed for 3 times with TBST for 10 min each. The membrane was probed with the following antibodies: β-actin (goat anti-mouse, 1:5000 dilution, Applygen), nPKCδ (goat anti-mouse, 1:5000 dilution, Santa Cruz), p-Akt (goat anti-rabbit, 1:5000 dilution, Santa Cruz), t-Akt (goat anti-rabbit, 1:5000 dilution, Santa Cruz), VCAM-1(goat anti-mouse, 1:5000 dilution, Santa Cruz). The membrane was incubated overnight at 4°C, after 3 times with TBST for 10 min each, membranes were incubated with HRP-labeled Goat Anti-Mouse IgG (1:5000 dilution, Applygen) or HRP-labeled Goat Anti-Rabbit IgG (1:5000 dilution, Applygen). The membranes were placed on a bleaching shaker for 1 h at room temperature. Developing Solution A and B were mixed in 1:1 ratio and dropped on membrane, then the PVDF membrane was immediately placed in a gel imager (Bio-Rad, United States) until the best band was appeared.

### Immunohistochemistry for VCAM-1 in the aortic root

In brief, 4 μm cross-section slides of aortic root were incubated with anti-VCAM-1 primary antibodies at 4°C overnight. After incubated with appropriate biotinylated secondary antibodies and HRP streptavidin, diaminobenzidine (DAB) was added for color development and hematoxylin for counterstaining. Images were collected on an Olympus FSX100 microscope (Olympus, Tokyo, Japan).

### Statistical analysis

The expression of each target protein was expressed semi-quantitatively by the ratio of the gray values of proteins (nPKCδ, p-Akt, t-Akt, and VCAM-1) to the gray values of the corresponding β-actin, and analyzed by the Image Lab analysis system. The independent experiment was repeated for 3 times. The statistical software package (SPSS for Window, Version 21.0, SPSS, United States) was used to analyzed the data. Measurement data was presented as mean value ± standard deviation (±SD). Comparisons among groups were performed using one-way ANOVA followed by the LSD test, and comparisons between two groups were performed using the two-sample t-test. A *P* value < 0.05 was considered statistically significant.

## Results

### Subject characteristics

A total of 40 CHD patients with a mean age of 48.28±10.11 years and a mean dialysis period of 44.17±25.11 months were enrolled in this study. The baseline demographic, clinical, biochemical characteristics of patients were described as shown in Table 1. The characteristics of patients with and without plaque were described as shown in Table 2.

**TABLE 1:** Characteristics of patients.

Items	Patients (n=40)
Age, years	48.28±10.11
Gender, male, n (%)	20 (50%)
Primary disease	
Chronic glomerulonephritis, n (%)	13 (32.50%)
Hypertensive nephropathy, n (%)	5 (12.50%)
Diabetic nephropathy, n (%)	9 (22.50%)
Chronic interstitial nephritis, n (%)	2 (5.00%)
Polycystic kidney disease, n (%)	2 (5.00%)
Unknow, n (%)	9 (22.50%)
Dialysis duration, months	44.17±25.11
Smoking, n (%)	5 (12.50%)
Diabetes, n (%)	9 (22.50%)
Kt/V	1.35±0.03
Hb, g/L	113.70±14.89
Alb, g/L	36.63±3.56
ALT, U/L	14.83±5.54
AST, U/L	14.05±4.49
TG, mmol/L	1.67±0.95
Tch, mmol/L	4.23±0.97
LDL-C, mmol/L	2.23±0.74
hsCRP, mmol/L	2.64±1.41
Cr, μmol/L	915.90±126.58
BUN, mmol/L	26.15±3.60
Ca, mmol/L	2.26±0.12
P, mmol/L	1.85±0.26
iPTH, pg/ml	254.43±75.47

Values are means ± SD, unless specified otherwise.

Hb: hemoglobin; Alb: albumin; ALT: alanine transaminase; AST: aspartate aminotransferase; TG: triglyceride; Tch: total cholesterol; LDL-C: low density lipoprotein-cholesterol; hsCRP: high sensitivity C reactive protein; Cr: creatinine; BUN: blood urea nitrogen; Ca: calcium; P: phosphorus; iPTH: intact parathyroid hormone; RASI: renin angiotensin system inhibitor; CCB: calcium channel blocker; β-blocker: β-receptor blocker.

**TABLE 2:** Characteristics of CHD patients with and without plaque.

Items	CHD (n=20)	CHD+AS (n=20)	t/χ2 value	*P* value
Age, years	46.85±10.70	49.70±9.55	0.889	0.380
Gender, male/female	9/11	11/9	0.400	0.527
Dialysis duration, months	43.24±23.49	45.10±27.21	0.232	0.818
Smoking, n (%)	3 (15.00%)	2 (10.00%)	0.229	0.633
Diabetes, n (%)	3 (15.00%)	6 (30.00%)	1.290	0.256
Kt/V	1.35±0.02	1.34±0.03	0.857	0.397
Hb, g/L	113.45±13.87	113.95±16.20	0.105	0.917
Alb, g/L	37.11±3.55	36.14±3.59	0.859	0.396
ALT, U/L	14.50±5.12	15.15±6.05	0.367	0.716
AST, U/L	14.10±4.85	14.00±4.23	0.069	0.945
TG, mmol/L	1.69±1.05	1.65±0.86	0.135	0.893
Tch, mmol/L	4.25±1.11	4.22±0.84	0.109	0.914
LDL-C, mmol/L	2.19±0.59	2.27±0.89	0.330	0.743
hsCRP, mmol/L	2.47±1.22	2.80±1.59	0.755	0.455
Cr, μmol/L	928.16±142.77	903.64±110.41	0.608	0.547
BUN, mmol/L	26.92±3.19	25.38±3.89	1.368	0.179
Ca, mmol/L	2.26±0.13	2.27±0.13	0.100	0.921
P, mmol/L	1.81±0.25	1.89±0.27	0.922	0.363
iPTH, pg/ml	270.11±77.84	238±71.51	1.327	0.192

Values are means ± SD, unless specified otherwise.

Hb: hemoglobin; Alb: albumin; ALT: alanine transaminase; AST: aspartate aminotransferase; TG: triglyceride; Tch: total cholesterol; LDL-C: low density lipoprotein-cholesterol; hsCRP: high sensitivity C reactive protein; Cr: creatinine; BUN: blood urea nitrogen; Ca: calcium; P: phosphorus; iPTH: intact parathyroid hormone; RASI: renin angiotensin system inhibitor; CCB: calcium channel blocker; β-blocker: β-receptor blocker.

### Activation of nPKCδ in CHD patients and CHD+AS patients

**FIGURE 1:**
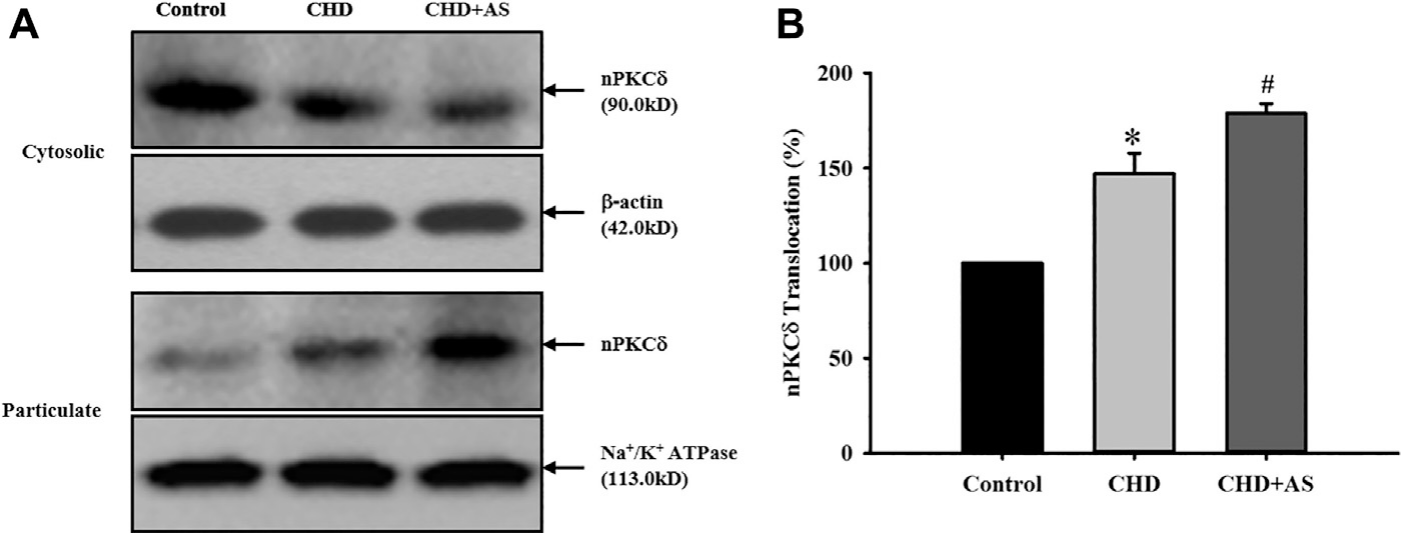
Activation of nPKCδ in CHD patients and CHD+AS patients. The membrane translocation of nPKCδ in CHD group was higher than that in control group (*P<0.05 vs. Control group), and the increase of nPKCδ membrane translocation in CHD+AS group was more obvious (^#^ *P*<0.05 vs. CHD group).

The activation of nPKCδ in CHD group was higher than that in control group, and the increase of nPKCδ membrane translocation in CHD+AS group was more obvious (Figure 1).

### CSE/H_2_S regulates activation of nPKCδ against formation of UAAS in mouse aorta (Figure 2)

**FIGURE 2:**
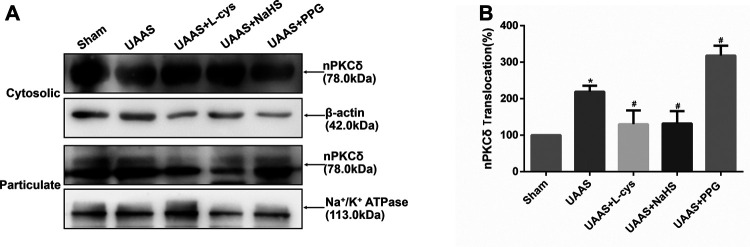
Effects of CSE/H2S system on the membrane translocation of nPKCδ in mouse aorta. The membrane translocation of nPKCδ in Sham, UAAS, UAAS+L-cys, UAAS+NaHS and UAAS+PPG group. **(A)** The protein contents in cytosolic and particulate fraction of mouse aorta were tested by Western blot; **(B)** Quantitative analysis showed that nPKCδ membrane translocation in UAAS group increased significantly compared with Sham group (*P<0.05 vs. Sham group, n=6 per group). UAAS+L-cys and UAAS+NaHS group decreased significantly compared with UAAS group, UAAS+PPG group increased significantly compared with UAAS group (^#^ *P*<0.05 vs. UAAS group, n=6 per group).

Aortic cytoplasmic protein and membrane related proteins were extracted, and Western blot detection showed that the activation of nPKCδ in UAAS group was higher than that in Sham group, and the difference was statistically significant (*P*<0.05, n=6). UAAS+NaHS group and UAAS+L-cys group were lower than UAAS group, and the difference was statistically significant (*P*<0.05, n=6). UAAS+PPG group was higher than UAAS group, and the difference was statistically significant (*P*<0.05, n=6).

### CSE/H_2_S regulates Akt phosphorylation against UAAS formation in mouse aorta (Figure 3)

**FIGURE 3:**
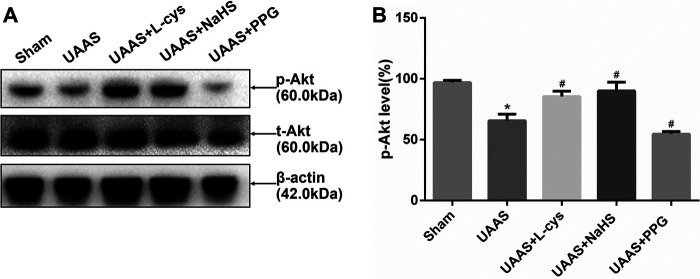
Effects of CSE/H_2_S system on the phosphorylation level of Akt in mouse aorta. The phosphorylation level of Akt in Control, Sham, UAAS, UAAS+L-cys, UAAS+NaHS and UAAS+PPG group. **(A)** The protein contents in mouse aorta were tested by Western blotting; **(B)** Quantitative analysis showed that Akt phosphorylation in UAAS group decreased significantly compared with Sham group (*P<0.05 vs. Sham group, n=6 per group). UAAS+L-cys group and UAAS+NaHS group were increased significantly compared with UAAS group, UAAS+PPG group decreased significantly compared with UAAS group (^#^ *P*<0.05 vs. UAAS group, n=6 per group).

Total aortic protein was extracted from mice, and Western blot detection showed that Akt phosphorylation level in UAAS group was lower than that in Sham group, and the difference was statistically significant (*P*<0.05, n=6). UAAS+L-cys group and UAAS+NaHS group were higher than UAAS group, and the difference was statistically significant (*P*<0.05, n=6). UAAS+PPG group was lower than UAAS group, and the difference was statistically significant (*P*<0.05, n=6).

### CSE/H_2_S system regulates VCAM-1 expression in mouse aorta against UAAS formation in mice aorta (Figure 4 and Supplementary Figure S1)

**FIGURE 4:**
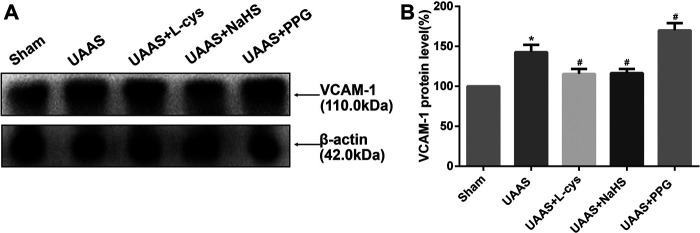
Effects of CSE/H_2_S system on the expression of VCAM-1 in mouse aorta. The expression of VCAM-1 in Control, Sham, UAAS, UAAS+L-cys, UAAS+NaHS and UAAS+PPG group. **(A)** The protein contents in mouse aorta were tested by Western blotting; **(B)** Quantitative analysis showed that VCAM-1 expression in UAAS group increased significantly compared with Sham group (*P<0.05 vs. Sham group, n=6 per group). UAAS+L-cys and UAAS+NaHS group decreased significantly compared with UAAS group, UAAS+PPG group increased significantly compared with UAAS group (^#^ *P*<0.05 vs. UAAS group, n=6 per group).

Total aortic protein was extracted from the aorta of mice, and the expression of VCAM-1 protein in UAAS group was higher than that in Sham group, with statistically significant difference (*P*<0.05, n=6). UAAS+ L-cys group and UAAS+NaHS group were lower than UAAS group, and the difference was statistically significant (*P*<0.05, n=6). UAAS+PPG group was higher than UAAS group, and the difference was statistically significant (*P*<0.05, n=6).

## Discussion

Cardiovascular disease is the most common complication and the leading cause of death in patients with chronic kidney disease, especially those on maintenance dialysis (Chirakarnjanakorn et al., 2017). In patients with end-stage renal disease, atherosclerosis begins earlier and progresses faster, which is called uremic accelerated atherosclerosis. In patients with end-stage renal disease, atherosclerosis is the main predictor of death from cardiovascular disease. To study the occurrence and progression mechanism of atherosclerosis in patients with end-stage renal disease is of great significance for the prevention and treatment of cardiovascular diseases. The pathogenesis of atherosclerosis includes vascular endothelial injury accompanied by dysfunction, monocyte infiltration, foam cell formation and vascular smooth muscle cell proliferation (Rahman and Woollard, 2017). In addition to traditional risk factors, dysregulation of calcium and phosphorus metabolism, anemia, oxidative stress, inflammation and other uremia related risk factors also play an important role in the occurrence and progression of atherosclerosis in patients with uremia (Christoffersen et al., 2017).

H_2_S is a newly discovered third gas signaling molecule after nitric oxide and carbon monoxide, which has a variety of physiological regulatory functions in the cardiovascular system (Wang et al., 2017). Endogenous H_2_S can be produced by enzymatic and non-enzymatic reaction pathways. It is mainly enzymatic reaction pathway in mammals, which is catalyzed by cystathionine-β-synthase (CBS), cystathionine-γ-lyase (CSE) and 3-mercaptopyruvate sulfurtransferase (3-MST) (Guan et al., 2012). Among them, L-cys was taken as the substrate for CBS and CSE, and β-mercaptopyruvate was taken as the substrate for 3-MST. The expression of three enzymes was tissue specific: nervous system mainly expressed CBS and 3-MST; cardiovascular system mainly expressed CSE. Both CBS and CSE are expressed in ileum mucosa, gastric mucosa, liver and kidney (Yang et al., 2008). Our previous study found that endogenous H_2_S production was reduced in maintenance hemodialysis patients, and endogenous H_2_S level was correlated with the risk of cardiovascular disease in patients with maintenance hemodialysis, and decreased plasma H_2_S level was a predictive factor of cardiovascular death in maintenance hemodialysis patients (Feng et al., 2015). Further experiments showed that exogenous H_2_S could inhibit the formation and progression of atherosclerosis in the UAAS model. Although the role of CSE/H_2_S system in UAAS has been preliminarily explored, its molecular mechanism has not been systematically elucidated.

PKC is a serine/threonine kinase involved in signal transduction, is the effector in the G protein coupled receptor system, in recent years, many studies have shown that PKC played a very important role in many cardiovascular system diseases such as atherosclerosis and vascular calcification, hypertension, myocardial ischemia-reperfusion injury, the occurrence of heart failure and arrhythmia development (Bynagari-Settipalli et al., 2010). According to the structure and sensitivity to Ca^2+^ and diacylgycerol (DAG), PKC can be divided into three categories: the conventional PKC (α, βI, βⅡ, γ), novel PKC (δ, ε, η, θ), atypical PKC (ζ, λ). PKC α, βI, βⅡ, γ and ε widely expressed in multiple tissues, whereas other subtypes showed tissue specificity. PKC and its associated signaling pathways are closely related to the formation and development of atherosclerosis. The results showed that inhibition of nPKCδ can increase the phosphorylation level of endothelial nitric oxide synthase (eNOS) and promote endothelial survival (Monti et al., 2010). What's more, nPKCδ knockdown could inhibit the macrophages from uptaking oxidized low-density lipoproteins, and foam cell formation (Lin et al., 2012).

Akt, also known as protein kinase B, belongs to serine/threonine protein kinase and has three subtypes in mammals: Akt1, Akt2 and Akt3, with tissue specific expression. Akt1 is widely expressed in various tissues. Akt2 is highly expressed in fat, liver and skeletal muscle. Akt3 is mainly expressed in brain and testis (Gonzalez and McGraw, 2009). Multiple studies have demonstrated that Akt plays an important role in multiple biological reactions, including cell survival, cell metabolism and transcriptional regulation (Yu and Cui, 2016). In the artery tissue, Akt also has expression specificity in different cells. Endothelial cells and VSMCs mainly expressed Akt1 subtype. In endothelial cells, Akt1 could promote cell survival by activating eNOS and NF-κB. In VSMCs, Akt1 could promote FOXO3a and GSK3 expression thus inhibiting cell apoptosis (Allard et al., 2008). In endothelial cells and VSMCs, Akt1 could inhibit the formation of atherosclerosis and play a protective role. In macrophages, Akt2 may be involved in inducing inflammatory response and foam cell formation, while Akt3 inhibits foam cell formation (Yu et al., 2015).

In the rat model of diabetes, increased activation of nPKCδ inhibits Akt phosphorylation and promotes retinal neuronal apoptosis (Kim et al., 2008), while increased NADPH oxidase activity inhibits nPKCδ activation and increased Akt phosphorylation in the rat model of stroke (Cai et al., 2017). Our previous study found that in dialysis patients with atherosclerosis, endogenous H_2_S were decreased compared with dialysis patients without atherosclerosis, with increased activation of peripheral mononuclear cells nPKCδ and decreased phosphorylation of Akt. These studies suggest that metabolic abnormalities of H_2_S are involved in the progression of uremia with cardiovascular disease, which may be mediated by PKC-related signaling pathways.

VCAM-1 is a pro-atherosclerotic adhesion molecule that plays an important role in the occurrence and development of atherosclerosis and plaque instability, and can promote leukocyte recruitment of atherosclerotic plaques (Radecke et al., 2015). Studies have shown that reduced Akt phosphorylation level in rat arterial endothelial cells can increase VCAM-1 expression (Pott et al., 2016).

ApoE^−/−^ mice are ideal animal models for hyperlipidemia and atherosclerosis, and their vascular pathophysiological changes are similar to human (Jawien et al., 2004). In our study, ApoE^−/−^ mice were treated with 5/6 nephrectomy and given a high-fat diet to establish a mouse UAAS model. CSE substrate L-cys, H_2_S donor NaHS and CSE inhibitors PPG were given by intraperitoneal injection. After six weeks’ injection, mice aorta were taken and Western blot method was used to detect nPKCδ activation, Akt phosphorylation and VCAM-1 expression. It was found that compared with Sham group, nPKCδ membrane translocation was significantly increased in UAAS group, accompanied by decreased Akt phosphorylation level and increased VCAM-1 expression, suggesting that 5/6 nephrectomy combined with high-fat diet may affect the nPKCδ/Akt signaling pathway. Compared with the UAAS group, nPKC activation was decreased in the UAAS+L-cys group and the UAAS+NaHS group, accompanied by an increase in Akt phosphorylation level and a decrease in VCAM-1 expression, while nPKCδ activation was significantly increased in the UAAS+PPG group compared with the UAAS group, accompanied by a decrease in Akt phosphorylation level and an increase in VCAM-1 expression, suggesting that H_2_S supplementation can inhibit nPKCδ activation, thus affecting the nPKCδ/Akt signaling pathway. nPKCδ/Akt signaling pathway is involved in the formation of UAAS in mice mediated by imbalance of CSE/H_2_S system.

However, the specific inhibitor or agonist of nPKCδ/Akt signaling pathway has not been used in this experiment, and other signaling pathways may also be involved in this process. At present, the mechanism of CSE/H_2_S system imbalance in UAAS has not been fully clarified. Further experiments are needed to investigate whether there are other pathways in addition to the nPKCδ/Akt signaling pathway, and whether other downstream molecules in addition to VCAM-1 were influenced by the nPKCδ/Akt signaling pathway.

## Conclusion

Endogenous CSE/H_2_S system may protected against the UAAS via nPKCδ/Akt signal pathway. nPKCδ/Akt signaling pathway may be involved in the UAAS in mice by affecting the expression of downstream VCAM-1.

## Data Availability

The raw data supporting the conclusions of this article will be made available by the authors, without undue reservation.
