# Supplementary figures and images for: Hydrogen Sulfide Protects Against Uremic Accelerated Atherosclerosis via nPKCδ/Akt Signal Pathway
Source: Front Mol Biosci. 2021 Feb 9;7:615816. doi: 10.3389/fmolb.2020.615816 (PMC7903246; doi:10.3389/fmolb.2020.615816)

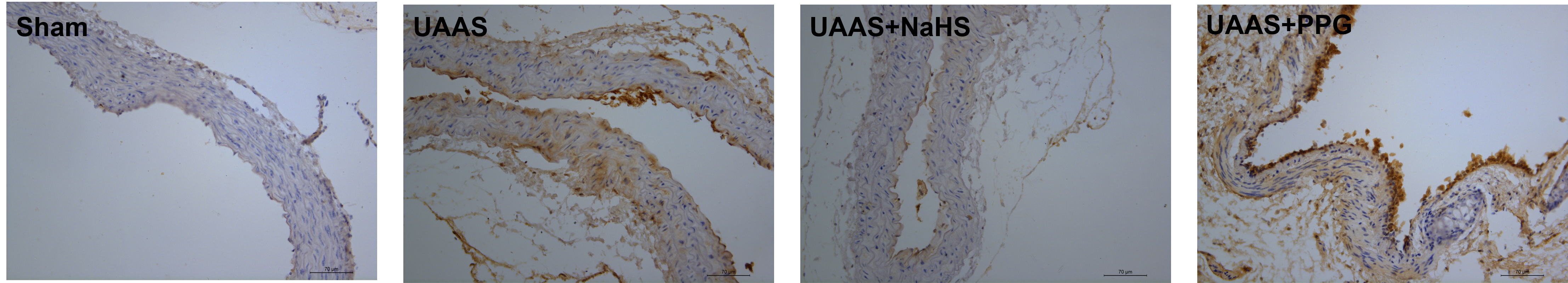

Supplement: Supplementary file 1 [file image1.tif]

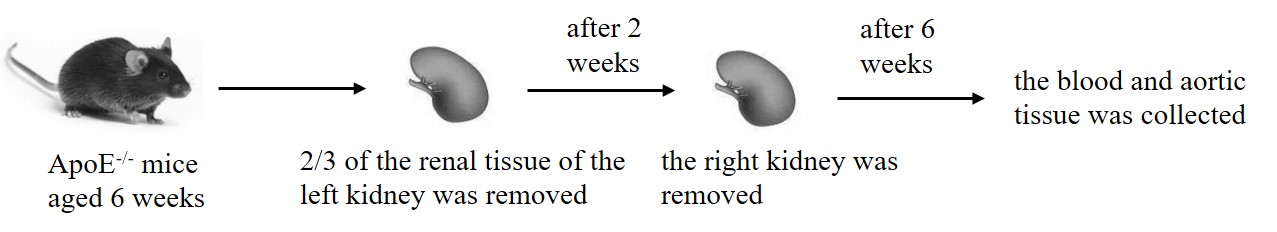

Supplement: Supplementary file 2 [file image2.jpeg]

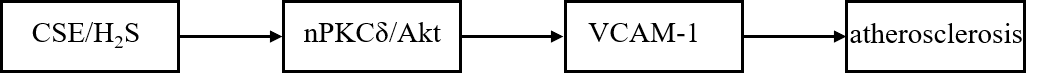

Supplement: Supplementary file 3 [file image3.tif]
